# Potential of Therapeutic Bacteriophages in Nosocomial Infection Management

**DOI:** 10.3389/fmicb.2021.638094

**Published:** 2021-01-28

**Authors:** Nannan Wu, Tongyu Zhu

**Affiliations:** ^1^Shanghai Institute of Phage, Shanghai Public Health Clinical Center, Fudan University, Shanghai, China; ^2^Shanghai Key Laboratory of Organ Transplantation, Zhongshan Hospital, Fudan University, Shanghai, China

**Keywords:** antimicrobial resistance, nosocomial infections, phage library, phage therapy, disinfection

## Abstract

Nosocomial infections (NIs) are hospital-acquired infections which pose a high healthcare burden worldwide. The impact of NIs is further aggravated by the global spread of antimicrobial resistance (AMR). Conventional treatment and disinfection agents are often insufficient to catch up with the increasing AMR and tolerance of the pathogenic bacteria. This has resulted in a need for alternative approaches and raised new interest in therapeutic bacteriophages (phages). In contrast to the limited clinical options available against AMR bacteria, the extreme abundance and biodiversity of phages in nature provides an opportunity to establish an ever-expanding phage library that collectively provides sustained broad-spectrum and poly microbial coverage. Given the specificity of phage-host interactions, phage susceptibility testing can serve as a rapid and cost-effective method for bacterial subtyping. The library can also provide a database for routine monitoring of nosocomial infections as a prelude to preparing ready-to-use phages for patient treatment and environmental sterilization. Despite the remaining obstacles for clinical application of phages, the establishment of phage libraries, pre-stocked phage vials prepared to good manufacturing practice (GMP) standards, and pre-optimized phage screening technology will facilitate efforts to make phages available as modern medicine. This may provide the breakthrough needed to demonstrate the great potential in nosocomial infection management.

## Introduction

Nosocomial infections (NIs), also known as hospital-acquired infections (HAI), are infections that are newly acquired in a hospital or other healthcare facility. They arise from various sources including healthcare staff, contaminated equipment, bed linen, and air droplets. NIs represent the most frequent adverse events during care delivery and typically result in prolonged hospital stays. There can be massive additional costs for health systems and patients, and unnecessary deaths (Allegranzi et al., 2011).

The misuse and overuse of antibiotics are accelerating the creation of antimicrobial resistance (AMR), which has become a major public health problem. In 2017, the World Health Organization (WHO) highlighted, as a global priority, a list of multidrug-resistant (MDR) bacterial pathogens (informally termed “superbugs”) to help in prioritizing the research and development of new and effective antibiotic treatments (World Health Organization, 2017). These healthcare-associated superbugs usually cause nosocomial outbreaks and opportunistic infections in hospitalized patients. The most prominent causative agents are carbapenem-resistant *Enterobacteriaceae* (CRE), carbapenem-resistant *Acinetobacter baumannii* (CRAB), MDR *Pseudomonas aeruginosa*, methicillin-resistant *Staphylococcus aureus* (MRSA), and vancomycin-resistant *Enterococcus* (VRE). In the absence of effective treatment for bacterial infections that have AMR, modern medical care becomes inadequate and the infections persist in the body, increasing the risk of spread to others. Even worse, increasing global connectivity facilitates the rapid transport of infectious agents and their resistance genes through the world (Mellon et al., 2020).

NIs pose a high healthcare burden in both developed and developing countries (Allegranzi et al., 2011), this problem is further aggravated by the global spread of multidrug-resistant organisms (MDROs), complicating infection management, limiting therapy options and resulting in poorer outcomes (Laxminarayan et al., 2013; Chng et al., 2020). The increasing AMR in bacteria has entailed a need for alternative approaches to therapy and prophylaxis of these infections and raised new interest in therapeutic bacteriophages (phages) for these purposes. Phages are viruses that exclusively infect bacteria and were used therapeutically against bacterial diseases almost immediately after their discovery. The extreme abundance and biodiversity of phages in our surroundings and inside our bodies constitute a source of natural precision agents for bactericidal use, which might play a major role in tackling the global AMR crisis. Despite the remaining obstacles for phage therapy such as regulatory issues and bacterial anti-phage resistance, efforts to make it available as modern medicine are ongoing. In fact, the potential of phages in clinical practice goes beyond therapeutic applications, it could be involved in the whole process of nosocomial infection management. In this review, we discuss the potential of therapeutic phages in nosocomial infection monitoring, phage therapy, and environmental disinfection. We also propose a practical model of nosocomial infection management that uses phages.

## Establishing a Phage Library With Broad-Spectrum and Poly Antimicrobial Coverage

A key precondition in phage application is an eligible phage library (phage bank) with broad-spectrum, poly antimicrobial properties, where many well-characterized phages are constantly collected and arranged according to the host species. Although an increasing number of phage libraries has been established recently, the overall scale is small and the information in most of the existing phage collections is incomplete (Table 1) (Yerushalmy et al., 2020). This strongly limits wide access to phage therapy since not all patients can afford the time-consuming process of mailing pathogenic bacteria from a healthcare facility to a phage repository, followed by screening lytic phages and mailing therapeutic phages back; neither can they afford time for *de novo* phage screening from nature. For this reason, both the quantity and quality of phage libraries need enhancement.

**TABLE 1:** Phage libraries worldwide*.

**Organization**	**Region**	**Size**	**Targets**	**References**	**Representative phage therapy reports**	**Clinical trial**
**Bioresource Centers**						
The Felix d’Hérelle Reference Center for Bacterial Viruses	Canada	>400	A few dozen hosts	https://www.phage.ulaval. ca/en/phages-catalog/	Not applicable	Not applicable
German Collection of Microorganisms and Cell Cultures-Bacteriophages	Germany	415	A few dozen hosts	https://www.dsmz.de/collection/	Not applicable	Not applicable
The National Collection of Type Cultures-Bacteriophages	UK	>100	*Campylobacter, Staphylococcus, Streptococcus*	https://www.phe-culturecollections.org.uk/	Not applicable	Not applicable
American Type Culture Collection-Bacteriophages	US	∼500	A few dozen hosts	https://www.atcc.org/	Not applicable	Not applicable
**Non-profit organizations**						
George Eliava Institute-Phage and Strain Collection	Georgia	>1000	180 bacterial species of 44 genera	http://eliava-institute.org/bacterial-strain-and-phage-collection/	Hoyle et al., 2018; Leitner et al., 2020	NCT03140085
Phage Therapy Unit-Bacteriophage Collection	Poland	>850	15 genera of the most common bacterial human pathogens	http://www.iitd.pan.wroc.pl/en/OTF/ZasadyTerapiiFagowej.html	Rostkowska et al., 2020; Żaczek et al., 2020	NCT00945087
Center for Phage Technology	US	Unknown	Unknown	https://cpt.tamu.edu/phage-therapy-projects/	Schooley et al., 2017	Not applicable
Pittsburgh Bacteriophage Institute-Actinobacteriophage Database	US	>15000	Species from *Actinobacteria* phylum	https://phagesdb.org/phages/	Dedrick et al., 2019	Not applicable
Yale University-Phage library	US	Unknown	11 species/genera of the most common bacterial human pathogens	http://www.benjaminchanphd.com/	Chan et al., 2018	NCT04636554
Fagenbank Phage Collection	Netherland	Unknown	9 species of the most common bacterial human pathogens	https://www.fagenbank.nl/	Not found	Not found
Bacteriophage Bank of Korea	Korea	>1000	21 species of the most common bacterial human pathogens	http://www.phagebank.or.kr/	Not found	Not found
Shanghai Institute of Phage-SIPhage Library	China	>600	ESKAPE, *Mycobacterium*	http://www.sipmed.cn/	Bao et al., 2020	ChiCTR1900020989, ChiCTR2000036801, ChiCTR2000037365
Hebrew University-Israeli Phage Bank	Israel	>300	16 different species	https://ronenhazanlab.wixsite.com/hazanlab/the-404-israeli-phage-bank	Nir-Paz et al., 2019	Not found
**Phage companies**						
Microgen-Bacteriophages Products	Russia	Unknown	The most common bacterial human pathogens	https://www.microgen.ru/en/products/bakteriofagi/	Sarker et al., 2017; McCallin et al., 2018	NCT04325685
Intralytix-Human phage therapy products	US	Unknown	*E. coli, Shigellosis*, Women’s health associated bacteria, *Enterococcus*	http://intralytix.com/index.php?page = hum	Not found	NCT03808103
Armata Pharmaceuticals	US	Unknown	The most common bacterial human pathogens	https://www.armatapharma.com/pipeline/pipeline-overview/	Schooley et al., 2017; Aslam et al., 2019; Maddocks et al., 2019	NCT04596319, NCT03395769, NCT02757755, ACTRN12616000002482
Locus Biosciences	US	Unknown	Multiple bacterial human pathogens	https://www.locus-bio.com/#pipeline	Not found	NCT04191148
Adaptive phage therapy-Phage Bank	US	Unknown	ESKAPE, *Burkholderia cepacia*	https://www.aphage.com/science/	Schooley et al., 2017; Aslam et al., 2019; Duplessis et al., 2019	NCT04636554, NCT04287478
Pherecydes Pharma	France	Unknown	Multiple bacterial human pathogens	https://www.pherecydes-pharma.com/	Ferry et al., 2018; Jault et al., 2019; Ferry et al., 2020	NCT02116010, NCT02664740
BiomX	Israel	Unknown	*Cutibacterium acnes, K. pneumoniae, P. aeruginosa, S. aureus, F. nucleatum*	https://www.biomx.com/pipeline/	Not found	BX001 against acne Phase I completed under cosmetic route
Phagelux	China	Unknown	*K. pneumoniae, P. aeruginosa, Staphylococcus, P. Acnes*	http://www.phagelux.com/	Not found	NCT04287478

*Data isolated from the corresponding website up to 30th Nov, 2020; Armata Pharmaceuticals was merged between C3J and AmpliPhi in 2019. The Clinical trial numbers beginning with NCT refer to clinical trials registered on ClinicalTrials.gov (https://clinicaltrials.gov/), ChiCTR trials registered on Chinese Clinical Trial Registry (http://www.chictr.org.cn/), ACTRN trials registered on Australian New Zealand Clinical Trial Registry (https://www.anzctr.org.au/).

A first step to build a phage library is to prepare a library of pathogenic bacteria that provides the baits for phage hunting. Phages can be isolated from diverse environmental samples, however, not all phages are useful for therapeutic use. Criteria of eligible phage candidates have been extensively discussed and, although estimates of index values vary among groups, there’s a general consensus that the key features that must be characterized include: host species, complete genome sequence (to exclude or to allow modification of phages with known integration/toxic/resistance genes), host range testing, and high lytic activity (Gill and Hyman, 2010; Letarov and Kulikov, 2018; Merabishvili et al., 2018). Some other factors such as high yield, identified receptor, morphology, anti-biofilm activity, low resistance induction, stability during storage, and pre-clinical evaluation (e.g., safety, efficacy, pharmacodynamics and pharmacokinetics, human immune responses) are generally considered to be important during optimization of phages for therapeutics (Malik et al., 2017; D´abrowska and Abedon, 2019; Hesse and Adhya, 2019; Kortright et al., 2019; Yerushalmy et al., 2020).

In contrast to the limited number of antibiotics available, the extreme abundance and biodiversity of phages in nature make it possible to build an ever-expanding phage library that collectively provides sustained broad-spectrum and poly microbial coverage against the most common nosocomial pathogens such as the ESKAPE group (*E. faecium, S. aureus, K. pneumoniae, A. baumannii, P. aeruginosa*, and *Enterobacter* species) (Rice, 2008). However, given the diversity of phage-host interactions, isolation of suitable phages for some group of pathogenic bacteria has been found to be difficult (Mattila et al., 2015). For instance, almost all known phages against *Helicobacter Pylori* and *Clostridia difficile* are temperate (Hargreaves et al., 2015; Muñoz et al., 2020). Phage engineering and technical improvement on new phage isolation will be helpful to overcome these obstacles. The collaboration and sharing of phage libraries initiated by the Phage Directory (https://phage.directory) are also conducive to pool a global-scale phage library and to connect demands for therapeutic phages.

## Constructing a Nosocomial Infection Monitoring Database by Using Phage Susceptibility Profiles

Rapid tracking of pathogen sources with appropriate subtyping tools and immediate initiation of appropriate antibacterial treatment are crucial in the management of nosocomial infections. However, conventional bacterial genotyping methods such as pulse-field gel electrophoresis (PFGE), multi-locus variable tandem repeat analysis (MLVA), multilocus-sequence typing (MLST), and whole-genome sequencing (WGS) are time-consuming, normally needing more than three workdays to yield results. In contrast, bacterial phenotypic technics such as antibiotic susceptibility test, immunological methods (serotyping), or phage-typing are limited in discriminatory power but advantageous to save time (Table 2; Ferrari et al., 2017; Quainoo et al., 2017; Sloan et al., 2017; Vijayakumar et al., 2019). For the most common nosocomial pathogens which grow fast, a phage-typing can be achieved within six to eight hours by phage susceptibility testing.

**TABLE 2:** Overview of common bacterial subtyping methods*.

**Method**	**Application scenarios**	**Subtype discrimination accuracy**	**Time to results from a colony**	**Commercial availability**	**Relative cost**
**Phenotypic subtyping**					
Antibiotic susceptibility test	To select effective antimicrobial drugs.	Worse than DNA-based methods.	1–2 days	Yes (Laboratory medicine)	Low
Phage susceptibility test	To select effective lytic phages.	May perform better than PFGE/MLST for some bacteria but worse for others.	1–2 days	No*	Medium
Immunological test	To applied for the preliminary identification.	Typically worse than DNA-based methods.	<1 day	Yes (Laboratory medicine)	Medium
**DNA-based subtyping**					
Pulsed-field gel electrophoresis (PFGE)	To subtype bacterial species causing infection outbreaks	Gold-standard for many different bacteria	3–4 days	Yes	High
Multiple locus variable number of tandem repeats analysis (MLVA)	Usually been performed for more subtyping details after PFGE.	May perform better than PFGE for some bacteria but worse for others	1–2 days	No	Medium
Multilocus sequence typing (MLST)	To characterize bacterial populations at larger geographic and temporal scales	Typically worse than PFGE	>3 days (send out sequencing)	Yes	High
Whole-genome sequencing (WGS)	To cluster isolates for bacterial outbreak analysis.	Best discrimination among molecular subtyping approaches	2–4 weeks (send out sequencing)	Yes	High

*Mayo Clinic Laboratories (US) and Dr Dangs Lab (India) have announced to commercialize phage susceptibility test in 2020 (Adaptive Phage Therapeutics, 2020a; ANI, 2020).

A phage susceptibility test is a method followed by bacterial diagnosis, to simultaneously test the contents of a sub-library (specific to the target bacterial species) of phage candidates against the bacteria isolated from a patient, thereby identifying phages for patient-specific precision therapy. Laboratory-developed phage susceptibility tests are widely used for therapeutic phage screening (Aslam et al., 2019; Dedrick et al., 2019; Kuipers et al., 2019; Nir-Paz et al., 2019; Bao et al., 2020; Petrovic Fabijan et al., 2020). Additionally, given the specificity of phage-host interactions, the distinguished phage susceptibility profiles can enable rapid fingerprinting of bacterial substrains. Several laboratories have applied this method to investigate outbreaks caused by nosocomial or foodborne bacterial pathogens (Demczuk et al., 2003; Boxrud et al., 2007; Cooke et al., 2010; Barco et al., 2013; Wuyts et al., 2015). However, due to the variable sizes and components among phage libraries, phage-typing is limited in discriminatory power than genotyping approaches. It needs standardization to guarantee comparability among laboratories, as well as methodology optimization to reduce labor costs (Ferrari et al., 2017).

Nevertheless, on the basis of known epidemiological situations and pre-established phage collections, phage susceptibility testing may serve as a rapid and cost-effective method for bacterial subtyping to routinely monitor nosocomial infections. It may also be a prelude to preparing ready-to-use phages for patient treatment and environmental sterilization. Data from several preliminary studies have shown good concordance of profiles between phage-typing and other subtyping methods in distinguishing bacterial isolates (Demczuk et al., 2003; Crabb et al., 2019; Sundell et al., 2019). However, the possibility of frequent shifts in phage susceptibility profiles of phage-resistant strains should be allowed for, especially by the medical establishments with an ongoing phage therapy practice. Nevertheless, a database of routine phage-typing profiles helps by providing an expandable reference map that can be adjusted based on conventional molecular genotyping methods. With further improvements in the phage library diversity and in understanding phage-bacteria interaction, hospital-wide surveys employing the phage-typing database will be increasingly feasible, providing valuable information for infection control and eventually becoming part of routine practice.

## Phage Therapy That Targets the Problematic Bacterial Pathogens

Fixed composition (*prêt-à-porter*) or customized screened (*sur-mesure*), that is the question for therapeutic phage selection (Pirnay et al., 2011). Fixed composition, which is a stable and widely distributed phage preparation, is more adaptive for the modern pharmaceutical regulations than a customized screened phage preparation (*sur-mesure*). However, due to the high species/strain specificity of phages and the frequently evolving phage resistance in bacteria, it is difficult for a fixed phage cocktail to provide broad coverage on clinical strains. There have been some well-conducted clinical trials on the effectiveness of fixed phage cocktails but they have failed to obtain positive results (Markoishvili et al., 2002; Wright et al., 2009; Gindin et al., 2019; Jault et al., 2019; Leitner et al., 2020).

In parallel with full-scale clinical trials, lessons learned from *sur-mesure* case studies have been reported by numerous groups all over the world. These efforts have advanced medicinal phage technologies and highlighted safety and efficacy of phage therapy (Table 3; Luong et al., 2020; Pires et al., 2020). However, the *sur-mesure* approach is not well compatible with modern licensing processes. The European Medicines Agency (EMA) and the US Food and Drug Administration (FDA) placed phages under the medicinal (biologicals) product regulation; this means that marketing a phage product requires proof of safety and efficacy, also of quality by manufacture under Good Manufacturing Practice (GMP) regulations (Pires et al., 2020).

**TABLE 3:** Pros and cons of phage therapy.

	**Antibiotic therapy**	**Phage therapy**		
		**Pros**	**Cons**	**Solutions or optimizations**
Number of agents	Less than 30 antibiotics available	Expansible	Higher screening cost, Longer time between diagnosis and treatment	Pre-collected phage library and high-throughput screening technology
Antimicrobial spectrum	Broad spectrum	High species or strain specificity, limited impact on microbiome	Pathogenic bacterial strains are required to allow for a logical and customized phage screening	Phage cocktail, combined treatment with other antimicrobials
Anti-biofilms activity	Non/low effective	Some can penetrate and destroy biofilms	Phage’s anti-biofilm activity is very specific and need to be pre-tested	Combined use of biofilm-destroyed phages with different depolymerases, as well as with other anti-biofilm agents
*de novo* resistance	Normally occurs slowly during antibiotic treatment	Phage can co-evolve to infect resistant bacteria	Rapid evolution of bacterial anti-phage resistance	Phage cocktail, combined treatment with other antimicrobials
Dosing	Constant dose and course of treatment	Self-amplifying in target bacteria	Floating dose and course of treatment	Need to be standardized and quantified
Safety	Safe under rational use	Generally recognized as safe, do not target eukaryotic cell and can be eradicated by immune system	Relatively fewer evidence from strict clinical trials, Phage-neutralizing antibody induced	Substitute new lytic phages for a new course of treatment
Adverse effect	Multiple side effects (e.g., intestinal disorders, allergies, organ toxicity) have been reported	Side effects attributed to phage therapy have rarely been described	Removal of bacterial debris and toxins during production is prerequisite	Phages need to be produced under Good Manufacturing Practices facilities with similar regulations for pharmaceutical products
Efficacy to sensitive bacteria	Supported by standard clinical trials	Adequate evidences from case studies	Lack of large-scale clinical investigations	Large-scale of standard clinical trials are needed.
Regulatory pathway	Slow but rules-based development process to ensure safety and efficacy	Rapid discovery process	Require innovative regulations for approving and manufacturing	Approve qualified phage collections as drug components
Clinical acceptability	Widely accepted for infection prophylaxis and treatment	Phage lysis capacity uncorrelated with bacterial drug-resistance level	Typically used only as a last-resort treatment	Mass education, apply phages to prophylaxis, early intervention and environmental sterilization

The good news is that some small companies focused on phage-specific therapies are growing fast and some authorities have been open to rigorously controlled clinical phage applications. Despite the long way to go before general approval is reached for the use of phage therapy, recent progress in phage manufacturing and quality control has revitalized phage therapy worldwide. Notably, the FDA has approved several investigational phage therapy pipelines targeting prosthetic joint infection, urinary/genital tract infection, chronic wound infection, and secondary bacterial pneumonia under the Investigational New Drug (IND) allowance (Adaptive Phage Therapeutics, 2020b; Armata Therapeuticals, 2020; Intralytix, 2020; Phagelux, 2020).

## Phage Disinfection for Nosocomial Transmission Control

Good infection prevention practices such as hand hygiene, daily and terminal cleaning, and disinfection have been demonstrated to reduce the incidence of healthcare-associated infections (Ariza-Heredia and Chemaly, 2018; Mitchell et al., 2019). However, conventional disinfection methods such as ultraviolet and chemical agents (e.g., hydrogen peroxide, chlorine-derivatives) are often insufficient to cope with the increasing complexity and tolerance of the target bacteria. The application context and frequency are limited by the presence of the patient in the room. The concentration of chemical disinfectants needs to be constrained to a narrow range to balance the disinfection effectiveness and the level of harmful residuals and byproducts (Dong et al., 2019). In consequence, pathogenic bacteria can rapidly recolonize the environmental surface after disinfection (Laxminarayan et al., 2013; Weber et al., 2019).

Meanwhile, there has been great interest in developing novel sterilizing methods, such as “self-disinfecting” surfaces, continuous room disinfection with diluted chemical sterilant, and other reagents (Weber et al., 2019). Among these, the potential of phages in environmental disinfection has been long neglected but is now being applied in food processing workshops, livestock farms, and croplands (Holtappels et al., 2020; Pinto et al., 2020; Żbikowska et al., 2020). Phage disinfectants possess multiple advantages over conventional ones: first, phages are a very abundant microbial resource that holds great promise and options for targeting problematic bacteria such as those with multiple AMR or biofilm formation properties. Second, phages are species-specific viruses of prokaryotes and do not infect mammalian cells, which allows disinfection unlimited by frequency or by the presence of the patient in the room. Consequently, phage application has been proposed as a potential decontamination method for hospital surfaces and water systems (Jensen et al., 2015; Mathieu et al., 2019).

Recently, there has been a trickle of studies that demonstrated the efficacy of phages to rapidly decrease the load of pathogens commonly associated with nosocomial infections when they are present on different types of surfaces and aqueous systems in healthcare facilities (Ho et al., 2016; D’Accolti et al., 2019; Tseng et al., 2019). Ho et al reported an investigation of phage intervention in the intensive care unit (ICU) rooms. They applied 500 ml of customized phage-containing preparation with a concentration of 10^7^ PFU (plaque forming unit)/ml for a ∼ 27m^3^ room. A significant reduction in CRAB-associated NIs was observed when adding a single aerosolized phage treatment to the conventional chemical-based disinfection performed in ICU rooms at the patient discharge (Ho et al., 2016). D’Accolti et al added phages to probiotic-based sanitation (PCHS) in the Staphylococcal-contaminated bathrooms (∼4.5 m^2^, applied 2 × 10^8^ PFU phages for a multiplicity of infection ∼1000) of General Medicine wards, reporting a rapid and significant decrease in *Staphylococcus spp.* load on treated surfaces, up to 97% more than PCHS alone (D’Accolti et al., 2019). Tseng et al developed a chamber study model to analyze the potential of aerosolized phage BTCU-1 in reducing airborne *Mycobacterium smegmatis* in different multiplicity of infection (MOI) and aerosol generation time. The result showed that, MOI of 10 000 for 10 seconds significantly blocked recovery of *M. smegmatis* colony from the culture medium (Tseng et al., 2019).

Notably, several challenges to balance between benefits and costs of implementing phage disinfection cannot be neglected. In particular, storage condition and content of phage library depend on the stability and consumption of stocked phages; the absent of the quality control specification (QCS) for environmental applied phages should be addressed. It is certain that manufacture and QCS of phage disinfectants do not require as much as therapeutic phages (GMP-approved, endotoxin removal), however, this strategy needs mass consumption of phage and thus is production-capacity dependent. Although phages should not replace broad-spectrum agents in terminal disinfection, they offer great potential for continuous target-specific applications where the target is drug-resistant and antimicrobial chemicals are either relatively ineffective or their use would result in unintended detrimental consequences.

## A Practical Model of Using Phage in Nosocomial Infection Management

The use of phages is consistent with the need of nosocomial infection control to diminish both bacterial infection and transmission, as well as to relieve the overuse of antibiotics. However, little has been described regarding the systemic application of phages in infection management. Nevertheless, a similar concept has been successfully practiced in several other settings (e.g., food, animals, crops) (Holtappels et al., 2020; Pinto et al., 2020; Żbikowska et al., 2020), where a few phage-based products have been approved by the supervisory authorities in the United States, Canada, Israel, Europe, Australia and New Zealand (Pinto et al., 2020).

**FIGURE 1:**
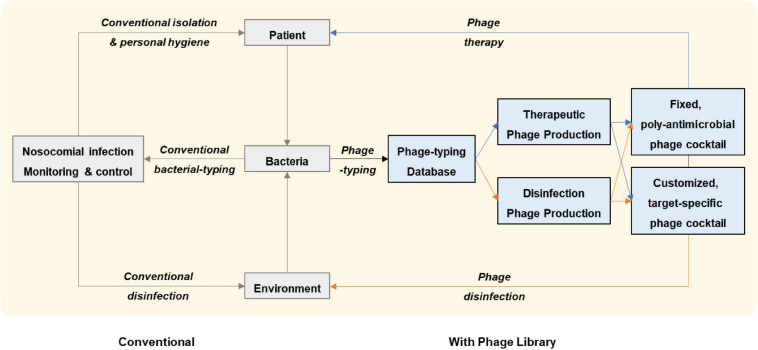
Workflow of nosocomial infection management proposed in this study. With an established phage library, lytic phages against a given pathogenic bacterium that has been isolated from a patient or from a hospital surface are screened and pre-stocked phage vials are delivered for application to the patient or to the environment. Retrospective and prospective phage susceptibility tests on hospital bacterial isolates can be united in a phage-typing database which can be further adjusted by conventional bacterial-typing methods and epidemiological links. Engaging the phage-typing database serves as a complementary strategy to conventional nosocomial infection management. By monitoring nosocomial infections in this way, fixed-composition phage cocktails with broad-spectrum, poly antimicrobial properties against the epidemic strains can be rapidly applied to the patient or the environment.

How might phages be integrated into the clinical workflow at a healthcare establishment? First, a system of cooperation between different departments (e.g., clinical, infectious diseases, laboratory, infection control, phage, pharmacy, and GMP factory) is essential. Second, an established phage library is needed for screening lytic phages against a given pathogenic bacterium isolated from a patient or from a hospital surface. Third, ready-to-use phage vials need to be properly manufactured prior to application to humans (GMP-approved) or environments; qualified phage vials should be pre-stocked to accelerate phage delivery. In parallel to use in phage therapy, the same phage formula can be aerosol-applied to the room or even the entire ward of the treated patient to prevent nosocomial transmission. Fourth, the accumulated phage-typing database of many bacteria can serve to monitor nosocomial infection and as a prelude to preparing phage stocks for patient treatment and environmental sterilization. Fifth, both retrospective and prospective phage susceptibility tests can be united (adjusted by conventional bacterial-typing methods and epidemiological links) to design fixed-composition phage cocktails with broad-spectrum, poly antimicrobial properties against the epidemic strains; the fixed-composition cocktails could then be applied in routine environmental sterilization or in treatment of serious infections such as septic shock or severe pneumonia that call for rapid initiation of treatment (Figure 1).

## Conclusion

The extreme abundance and biodiversity of phages in our surrounding environment and inside our body provides an opportunity to establish an ever-expanding phage library that collectively provides a sustainable broad-spectrum and poly microbial coverage. This library might play a major role in tackling the global AMR crisis. Whilst acknowledging the remaining obstacles for clinical phage application, we believe that by taking advantage of pre-established phage libraries, pre-stocked phage productions, and pre-optimized phage screening technology, phages can become available as modern medicine. This availability may constitute a breakthrough in both clinical treatment and environmental disinfection in the context of nosocomial infection management.

## Author Contributions

NW and TZ reviewed the literature, summarized the tables, conceptualized the figure, wrote the manuscript, and acquired funding. Both authors contributed to the article and approved the submitted version.

## Conflict of Interest

The authors declare that the research was conducted in the absence of any commercial or financial relationships that could be construed as a potential conflict of interest.
